# Effects of cold storage on double integrating sphere optical property measurements of porcine dermis and subcutaneous fat from 400 to 1100 nm

**DOI:** 10.1117/1.JBO.30.1.015001

**Published:** 2025-01-22

**Authors:** Maria A. T. Hoffman, Mark A. Keppler, Andrea L. Smith, Anjelyka Fasci, Matthew E. Macasadia, Amanda J. Tijerina, Robert Lyle Hood, Michael P. DeLisi, Joel N. Bixler

**Affiliations:** a SAIC, JBSA Fort Sam Houston, Texas, United States; b Texas A&M University, Department of Biomedical Engineering, College Station, Texas, United States; c University of Texas at San Antonio, Department of Mechanical Engineering, San Antonio, Texas, United States; d Conceptual Mindworks, Inc., San Antonio, Texas, United States; e Air Force Research Laboratory, JBSA Fort Sam Houston, Texas, United States

**Keywords:** skin properties, double integrating spheres, tissue storage, tissue preparation, inverse adding-doubling method, optical properties

## Abstract

**Significance:**

Accurate values of skin optical properties are essential for developing reliable computational models and optimizing optical imaging systems. However, published values show a large variability due to a variety of factors, including differences in sample collection, preparation, experimental methodology, and analysis.

**Aim:**

We aim to explore the influence of storage conditions on the optical properties of the excised skin from 400 to 1100 nm.

**Approach:**

We utilize a double integrating sphere system and inverse adding-doubling approach to determine absorption, $\mu_{a}$, and reduced scattering, $\mu_{s}^{\prime }$, coefficients of the porcine dermis and subcutaneous fat before and after refrigeration, freezing, or flash freezing.

**Results:**

Our findings indicate a small average change of −0.005, −0.003, and $0.002\;{\mathrm{mm}}^{-1}$ in $\mu_{a}$ for the dermis and 0.001, −0.003, and $-0.008\;{\mathrm{mm}}^{-1}$ for the subcutaneous tissue after refrigeration, freezing, and flash freezing, respectively, with the most notable differences observed in the hemoglobin absorption region. The value of $\mu_{s}^{\prime }$ shows a negligible average change of −0.05, −0.001, and $-0.02\;{\mathrm{mm}}^{-1}$ for the dermis, and 0.06, −0.1, and $0.03\;{\mathrm{mm}}^{-1}$ change for the subcutaneous tissue for refrigerated, frozen, and flash-frozen samples, respectively.

**Conclusions:**

The results provide additional context for the variability of published values of optical parameters and enable informed selection of sample storage conditions for future measurements. In addition, the results discussed here can be used to improve study planning, particularly with regard to maximizing the use of finite samples that have been collected.

## 1 Introduction

In recent decades, remarkable progress has been made in utilizing lasers for medical applications, both in advancing diagnostic imaging systems and in therapeutic treatments.^1^ These advancements can be attributed, in part, to improvements in computational modeling,^2^^–^^4^ parallel processing,^5^ and, more recently, machine learning.^6^ Although computational models are becoming quite effective at predicting outcomes of laser-tissue interactions, these models are only as accurate as the physical measurements that inform them.^7^^,^^8^ More specifically, highly accurate measurements of the absorption coefficients, reduced scattering coefficients, and anisotropy of tissues are crucial, as these properties are known to be predictive of tissue morphology and biochemical composition.^9^

Over the years, numerous studies have explored techniques to characterize optical properties from a broad range of tissues, including the skin.^10^^–^^12^ Despite the substantial volume of published data on the optical properties of the skin, there is considerable variability in reported values.^13^^,^^14^ For instance, when comparing the absorption coefficient of the human skin at a wavelength of 1000 nm, a five-fold difference in the reported values is present. Bashkatov et al.^15^ noted a measurement of $0.027\;{\mathrm{mm}}^{-1}$, whereas Chan et al.^16^ reported a significantly higher measurement of $0.155\;{\mathrm{mm}}^{-1}$. Troy and Thennadil,^17^ utilizing a double integrating sphere setup, obtained a result of $0.098\;{\mathrm{mm}}^{-1}$. These disparities can be attributed to tissue’s inherent heterogeneity, dynamic nature, scattering complexity, non-uniform absorption, temperature sensitivity,^18^^,^^19^ and poro-elastic composition.^20^ In addition, the optical properties of tissues can be expected to change after excision from their *in vivo* environment due to processes such as desanguination, cell necrosis, protein denaturation, and changes in tissue hydration. Optical system constraints, analysis methodologies, and sample preparation further exacerbate these differences.

As access to fresh tissue samples is not always available or practical, some degree of disagreement may also be attributed to tissue storage prior to measurements. Practical constraints such as transportation, the necessity of aligning research around subject availability and researcher schedules, and precision slicing with a cryotome all require short- or long-term tissue preservation to maintain tissue composition. Various storage methods are employed for preserving tissue, each with distinct implications for its integrity. Refrigeration and freezing require little preparation and are widely used. Cryopreservation at −80°C can be employed without cryoprotectants or with additives such as dimethyl sulfoxide, resulting in controlled freezing that maintains structural properties.^21^ By contrast, lyophilization enables extended storage through water removal, though rehydration is necessary before analysis.^22^ Alternatively, glycerol preservation represents an alternative approach to tissue maintenance.^22^ Understanding these preservation methods’ effects on optical properties is necessary for establishing reliable experimental protocols and interpreting measured changes in tissue characteristics.

Of chief concern here is the degree to which the optical properties of tissues change when refrigeration or freezing is utilized. Few previous studies have explored the influence of storage temperatures on tissue optical properties. Salomatina and Yaroslavsky observed changes in optical properties of mouse ear samples within 5 to 10 min *post-mortem*, after 72 h refrigeration at 4°C, and after 72 h refrigeration followed by a 1-h freeze-thaw cycle.^23^ They noted a decrease in absorption coefficient, especially in the region dominated by the absorption of hemoglobin, and a small overall decrease in scattering coefficient for stored samples, compared to 5- to 10-min *post-mortem* measurements. Similarly, investigations by Roggan et al.^24^ and Mesradi et al.^25^ indicated reductions in absorption and reduced scattering coefficient values between fresh and frozen samples of the liver, brain, heart, and kidney tissues.

More recently, DeLisi et al. found marginal differences in optical properties of fresh, refrigerated, and frozen porcine dermis, epidermis, and subcutaneous fat using goniometric spectrophotometry and Monte Carlo-based lookup tables.^26^ Observations were confounded by large standard deviations and inherent limitations with the methodology used. More specifically, the goniospectrophotometry method suffered from low throughput, resulting in a low signal-to-noise ratio (SNR), as well as signal crossover between silicon and In-Ga-As detectors. The goniometric reconstruction method utilized a spline curve fit for determining the spatial signal distribution, and values for specular reflectance and regular transmittance were roughly estimated due to a small number of measured angles. In addition, the results were not validated against a stable standard, such as a calibrated tissue optical phantom. As such, despite the demonstration of marginal differences, critical temperature-dependent changes may have remained undetected.

The objective of the present investigation was to expand and improve upon DeLisi et al.’s goniospectrophotometry study and to analyze the effect of storage on optical properties using a double integrating sphere system. Double integrating spheres are a widely accepted standard for measurements of reflectance and transmittance at all scattering angles that avoids many of the aforementioned constraints of the goniometric approach. In addition, a stable polyurethane tissue optical phantom ensured reproducible system performance, and detector crossover was eliminated by utilizing extended near-infrared (NIR) range silicon spectrometers. The absorption, $\mu_{a}$, and reduced scattering, $\mu_{s}^{\prime }$, coefficients were calculated from measured sample reflectance and transmittance using the inverse adding-doubling (IAD) method.^27^ We investigated changes in these optical parameters in the 400 to 1100 nm range in *ex vivo* samples of excised Yucatan miniature pig dermis and subcutaneous fat after refrigeration, freezing, and flash freezing, compared to pre-storage values. Our findings indicate a small average change in the value of $\mu_{a}$, with an ∼−0.005, −0.003, and $0.002\;{\mathrm{mm}}^{-1}$ change for dermis and 0.001, −0.003, and $-0.008\;{\mathrm{mm}}^{-1}$ for subcutaneous tissue in refrigerated, frozen, and flash frozen groups, respectively. The most notable differences are observed in the region dominated by hemoglobin absorption, with spectra of pre-storage and refrigerated dermis indicating the presence of deoxyhemoglobin, whereas those of frozen and flash-frozen dermis and all subcutaneous tissue samples exhibiting features characteristic of oxyhemoglobin. The value of $\mu_{s}^{\prime }$ showed a decrease of ∼−0.05, −0.001, and $-0.02\;{\mathrm{mm}}^{-1}$ for the dermis, and mixed response of 0.06, −0.1, and $0.03\;{\mathrm{mm}}^{-1}$ change for subcutaneous tissue in refrigerated, frozen, and flash-frozen storage conditions.

## 2 Methods

### 2.1 Optical System

**Fig. 1:**
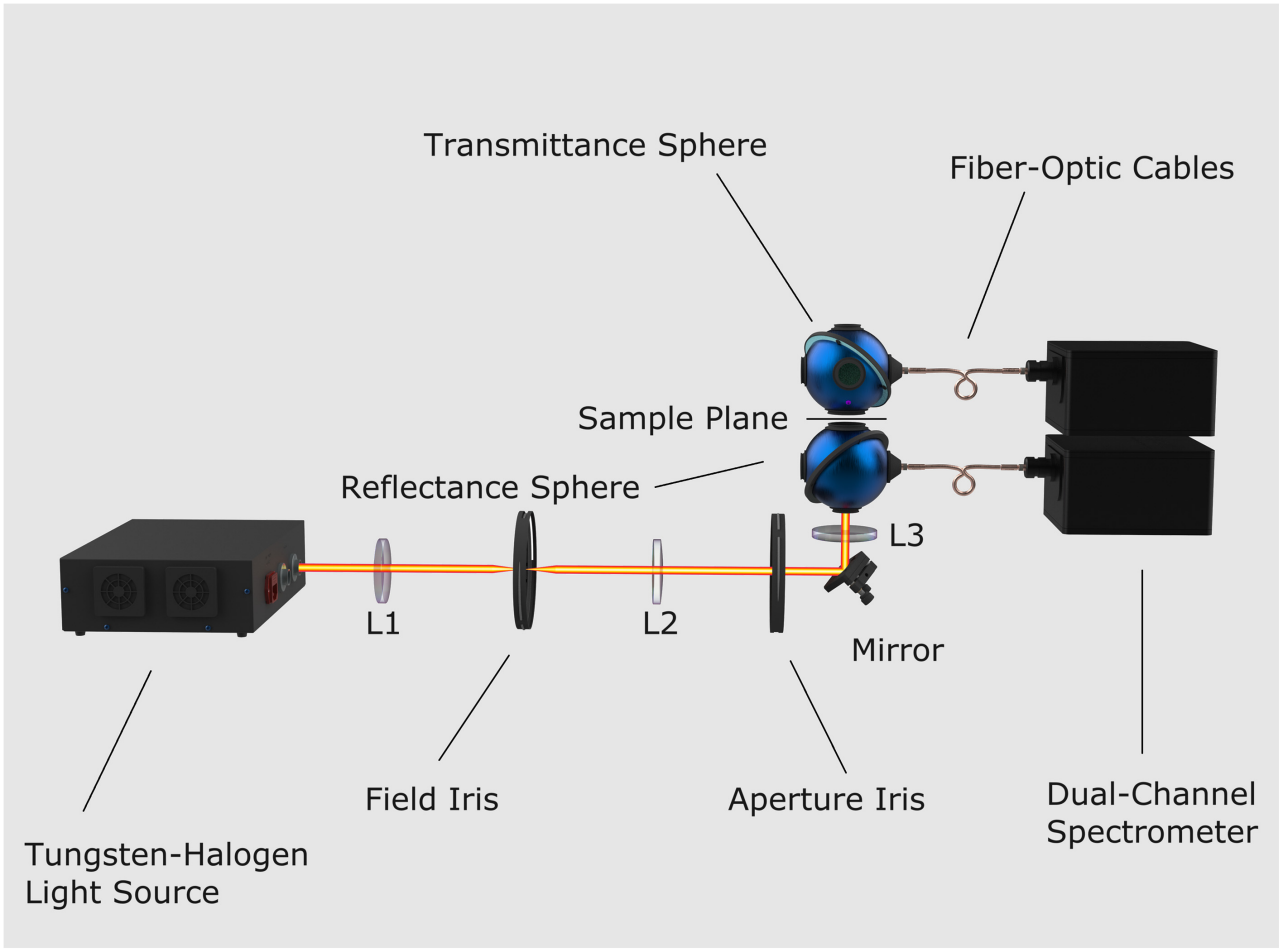
Double integrating sphere optical system employed a tungsten halogen light source equipped with an internal F/1 collector lens. A series of N-BK7 lenses was used to provide uniform illumination at the sample plane. A 38-mm FL biconvex lens (L1) acted as a condenser to increase fluence at the field iris. Two 150-mm FL plano-convex lenses (L2 and L3) imaged the field iris of the sample. The field iris, situated at an image plane complementary to the sample plane, enabled control of the spot diameter at the sample plane. The aperture iris, located at an image plane conjugate to the sample plane, served to attenuate the light incident on the samples. The 90-deg ports were linked to a dual-channel UV/VIS spectrometer via broadband fiber optic cables with 600  μm core diameters.

Total transmittance and reflectance of the porcine dermis and subcutaneous tissue were measured using a double integrating sphere system, shown in Fig. 1. Two identical four-port Spectralon^®^-coated integrating spheres (4P-GPS-033-SL, Labsphere, United States) were oriented such that their sample ports faced each other. The following detailed description of the layout uses the formal terminology of the ports, not their orientation in the system. Each sphere featured an interior diameter of 3.3″, and port diameters of 1″ for the 90 deg, 180 deg, and north pole ports. The sample (0 deg) port diameter was reduced using Spectraflect^®^-coated port reducer caps to 6.35 mm on both spheres. The spheres also included an internal baffle between 0 deg and 90 deg ports that prevented the directly reflected or transmitted light from reaching the detector. The sample, sandwiched between two fused silica slides, was placed between the two spheres’ sample ports. A custom-built enclosure covered both integrating spheres, providing a barrier against ambient light sources and external disturbances.

A broadband tungsten halogen lamp (66184, Oriel, United States) supplied the incident flux through the 180-deg port of the reflectance sphere. The illumination branch of the optical system included the lamp’s internal F/1 collector lens and a series of N-BK7 lenses to achieve uniform illumination at the sample plane. A 38-mm focal length (FL) biconvex lens (L1) acted as a condenser to increase the fluence at the field iris. A field iris in the illumination train controlled the spot size on the tissue, whereas an aperture iris at the conjugate image plane limited incident flux, preventing potential tissue heating. Two 150-mm FL plano-convex lenses in a 4f configuration (L2 and L3) projected an image of the field iris to the sample plane. All measurements were acquired with a 1-mm diameter spatially uniform spot at the sample plane.

Spectraflect^®^-coated port plugs (PP-100-SL, Labsphere, United States) covered the unused 180-deg port of the transmittance sphere and both north pole ports. The two 90-deg ports coupled in 600  μm-core, 0.22 NA optical fibers (FC-UVIR600-1-BX, Avantes) via SMA port caps (SMA-100-SF/SL, Labsphere, United States). The 200- to 2500-nm transmission range optical fibers transmitted the integrated reflection and transmission spectra to a dual channel spectrometer (Avaspec-ULS2048XL-2-EVO, Avantes, United States) equipped with a 500-nm blaze 300 lines/mm grating, 50  μm slit, order-sorting coating (OSC-UA, Avantes, United States), and a detector collection lens (DCL-UV/VIS-200, Avantes, United States).

The tungsten halogen lamp was given a stabilization period of no less than 30 min prior to the first measurement. Before any tissue measurements were acquired, the system baseline was established using a commercially sourced polyurethane Biomimic™ standard (F0660, INO, Canada). The accompanying Supplementary Material provides further information on the phantom and the measurements.

### 2.2 Tissue Preparation

Tissue samples were procured from a recently euthanized 3.3-month-old female Yucatan miniature pig, under a tissue-sharing protocol approved by the Institutional Animal Care and Use Committee (IACUC), which permitted *post-mortem* tissue sharing from pigs involved in other approved studies. The skin of female Yucatan miniature pigs within this age range is optically, physiologically, and morphologically similar to the human skin.^28^^,^^29^ In addition, the epidermis of the Yucatan mini-pig has melanin concentration and distribution that is more consistent with that of human skin, unlike other porcine breeds with less pigmentation, such as the commonly used Yorkshire pig.^30^ Although the epidermis layer was not considered in the present study, the selected animal model of the human skin makes the results particularly relevant to the research focused on laser exposure.

Tissue samples were prepared within 1 h post-euthanasia. Biopsies were taken from the flank of the deceased pig using a 10-mm disposable biopsy punch (P1025, Accuderm, United States), placed within a specimen bag, and submerged in an ice bath to increase sample rigidity during slicing, which was necessary to ensure consistent thickness. These punches were manually sectioned with a razor blade to isolate the dermis and subcutaneous fat layers under a dissection microscope. Immediately after sectioning, the samples were wrapped in saline-moistened gauze and placed in a Petri dish to maintain hydration until the pre-storage optical properties measurement was taken, for no more than 2 h. The average sample thickness before storage was 1.491±0.248  mm for the dermis tissue and 1.255±0.361  mm for the subcutaneous layer.

### 2.3 Tissue Storage and Measurement

The excised dermis and subcutaneous fat samples were allocated into one of three distinct storage conditions: refrigerated, frozen, or flash frozen, with ten samples per tissue type assigned to each group. The type and duration of storage were chosen to represent realistic laboratory scenarios: short-term refrigeration or longer-term storage between tissue removal and optical property measurements.

For each sample, a pre-storage optical properties measurement was taken within 2 h post-excision to establish a baseline. Just prior to that assessment, each sample was removed from the saline-moistened gauze and placed between two fused silica glass slides (#34-599, Edmund Optics, United States; each 0.95±0.06  mm thick) without compression, with the top slide simply resting on the sample. The sample thickness, a value required by the IAD algorithm, was determined using a dial thickness gauge (GA-725, Vigor, United States) before the assembly was placed into the double-integrating sphere system for analysis.

Following the pre-storage optical properties measurements, each sample was removed from the glass slides and securely enclosed within an individual plastic bag, with excess air expelled. The refrigerated group’s samples were additionally wrapped in saline-moistened gauze before being placed in the plastic bags. The orientation of all samples was marked and maintained in future measurements to ensure directional consistency of incident exposure to the anterior tissue surface. The refrigerated group was maintained in an environment of approximately 4°C for 24 h, whereas the frozen group was held at −18°C for 7 days. For the flash-frozen regimen, samples were immersed in liquid nitrogen for 30 s and then stored in a −80°C freezer for 7 days.

Prior to post-storage measurement, all samples were brought to room temperature. Sealed frozen and flash-frozen specimen bags were warmed in a bead bath incubator, avoiding direct contact with any surfaces, and set to 37°C for 10 min. Refrigerated specimen bags were maintained at room temperature for 10 min. Following this, samples were placed between two glass slides, with a drop of normal saline solution added to facilitate a consistent fluid interface between the glass windows and the sample as needed. Prior to each optical property measurement, sample thickness was re-taken to account for any storage-associated changes. The sample assembly was then positioned between the integrating spheres, with lateral adjustments made to ensure the focal spot was centered on the sample before data acquisition commenced.

### 2.4 Data Processing

The IAD method^27^ obtained the absorption and reduced scattering coefficients using Prahl’s IAD software, version 3.16.3.^31^ The IAD input file for each sample consisted of its normalized transmittance (1) and reflectance (2) at each wavelength, along with the parameters listed in Table 1. The sphere wall reflectance was determined as suggested in Ref. 31 using a 532-nm light source.

**Table 1:** Input parameters for the inverse adding doubling (IAD) algorithm, used to calculate the tissue absorption, $\mu_{a}$, and reduced scattering, $\mu_{s}^{\prime }$, coefficients. All parameters were constant for all samples except for sample thickness.

IAD parameter	Value
Beam diameter	1 mm
Sample thickness	Variable (mm)
Sample refractive index	$1.4^{12}$
Sample anisotropy	$0.9^{12}$
Top/bottom glass slide thickness	0.95 mm
Top/bottom glass slide refractive index	1.458
Sphere diameter	83.8 mm
Sample port diameter	6.35 mm
Entrance port diameter	25.4 mm
Detector port diameter	0.6 mm
Wall reflectance	0.95
Standard reflectance	0.39

Normalized transmittance as a function of wavelength, T(λ), was calculated using Eq. (1):^32^^,^^33^

$$T(\lambda )=\frac{S_{t}(\lambda )-N_{t}(\lambda )}{F_{t}(\lambda )-N_{t}(\lambda )},$$ (1)

where $S_{t}(\lambda )$ is the transmission measurement of the sample, $N_{t}(\lambda )$ is the null transmission signal, and $F_{t}(\lambda )$ is the full throughput of the transmittance sphere.

Likewise, the normalized reflectance, R(λ), was calculated by Eq. (2):

$$R(\lambda )=\rho (\lambda )\times \frac{S_{r}(\lambda )-N_{r}(\lambda )}{P_{r}(\lambda )-N_{r}(\lambda )},$$ (2)

where ρ(λ) is the reflectance of the calibration standard, a value provided by Avian Technologies, $S_{r}(\lambda )$ is the reflection measurement of the sample, $N_{r}(\lambda )$ is the null, or background, reflection signal, and $P_{r}(\lambda )$ is the reflection measurement of the calibration standard.

The four measurements for reflectance and transmittance normalization, $N_{t}(\lambda )$, $F_{t}(\lambda )$, $N_{r}(\lambda )$, and $P_{r}(\lambda )$, were acquired as outlined by Prahl et al.^32^^,^^33^ These calibration measurements were re-acquired before each storage group. As described earlier, Spectraflect^®^-coated port reducers were fitted over the sample port of each integrating sphere. To minimize single substitution error, the calibration reflectance standard was placed in a custom mount that ensured repeatable positioning that matched the offset of the sample relative to the sample port of the reflectance integrating sphere. The full throughput for the transmittance sphere was measured with the two spheres aligned and fit together with no sample within the sample port. For the transmittance and reflectance sphere’s null measurements, the aperture iris was covered with black anodized aluminum foil tape (T205-2.0, Thorlabs, United States). The reflectance sphere’s maximum throughput was recorded using a 40% reflectance standard (FSS-08-01c, Avian Technologies, United States), calibrated by Avian Technologies within a 2-year period.

## 3 Results and Discussion

**Fig. 2:**
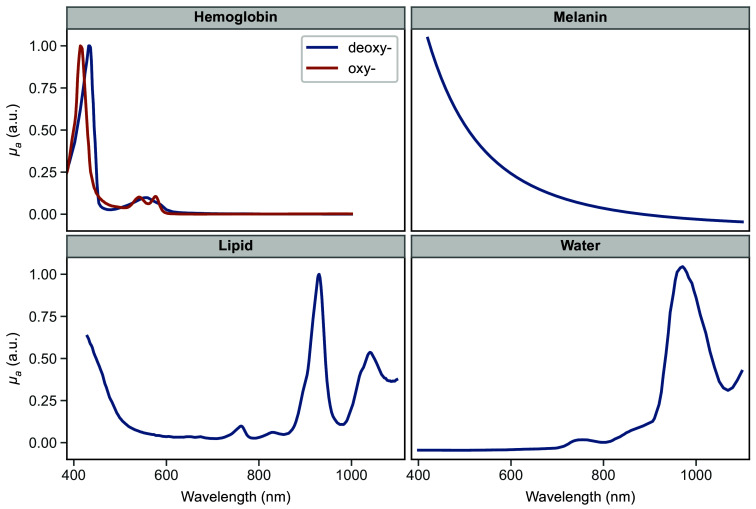
Representative normalized absorption of characteristic skin chromophores: oxy- and deoxyhemoglobin,^34^ melanin,^35^ lipid,^36^ and water.^37^ Axes are kept consistent.

The following section is subdivided into discussions of pre-storage measurements, the changes in absorption and reduced scattering coefficients following storage, and the assessment of methods. The average pre- and post-storage values of $\mu_{a}$ and $\mu_{s}^{\prime }$ over the 400 to 1100 nm range are available in the accompanying Supplementary Materials in 10 nm increments. To aid in the interpretation of the absorption spectra, representative absorption of characteristic skin chromophores is provided in Fig. 2. Please note that the representative spectra are normalized for ease of identification of characteristic peaks.

### 3.1 Pre-storage Optical Properties

**Fig. 3:**
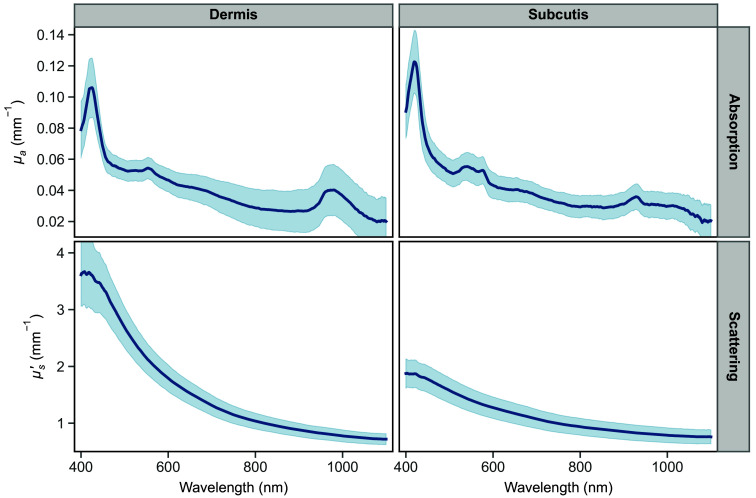
Average pre-storage absorption (top) and reduced scattering (bottom) coefficient values across all groups for dermis (left) and subcutaneous tissue (right). Shaded regions represent the standard deviation.

Figure 3 shows the measured pre-storage absorption, $\mu_{a}$, and reduced scattering, $\mu_{s}^{\prime }$, coefficient values for the dermis and subcutaneous layers, averaged across pre-storage measurements of all storage groups. For comparison, the present study’s recorded dermis $\mu_{a}$ and $\mu_{s}^{\prime }$ values fall within the range referenced in the introduction (for human dermis and epidermis):^15^^–^^17^ $0.037\;{\mathrm{mm}}^{-1}$ and $0.776\;{\mathrm{mm}}^{-1}$ at 1000 nm for $\mu_{a}$ and $\mu_{s}^{\prime }$, respectively.

The longer-wavelength end of the absorption spectrum is dominated by the water absorption with a peak at 975 nm in the dermis, but not the subcutaneous tissue. Small differences in dermis sample hydration likely explain the increased standard deviation in this region, compared with the rest of the spectrum. The absorption coefficient spectrum of subcutaneous fat has a noted presence of the lipids absorption peak around 928 nm, a characteristic chromophore for that tissue.^36^^,^^38^

Another region of interest is the 400 to 600 nm part of the $\mu_{a}$ spectrum, which is dominated by hemoglobin absorption. The absorption spectra of heme proteins are characterized by several strong absorption bands in that region: namely, the Soret band (also called B band) in the 400 to 440 nm range and one or two weaker Q bands in the 480 to 600 nm range. The number and location of these characteristic bands can be used to discriminate between oxyhemoglobin and deoxyhemoglobin.^34^^,^^39^^–^^41^ Deoxyhemoglobin is characterized by a 425-nm Soret band peak and a single Q band peak around 560 nm. With the transition to oxyhemoglobin, the Soret band experiences a subtle blue shift to about 415 nm, and two distinct Q band peaks are present at 540 and 578 nm.

As seen in Fig. 3, the average $\mu_{a}$ for the dermis has features indicative of deoxyhemoglobin, whereas the subcutaneous tissue has absorption peaks that correspond to oxyhemoglobin. The relatively higher concentration of oxyhemoglobin found in subcutaneous tissue as compared with the dermis could be attributed to the higher metabolic activity within the dermis. The oxygen expenditure of cells within the excised dermis would then result in the conversion of oxyhemoglobin into its deoxygenated form, whereas the lower metabolism within the subcutaneous fat would have a reduced effect on the level of oxyhemoglobin. In addition, Tseng et al. demonstrated in *in vivo* human skin measurements that higher quantities of oxyhemoglobin were detected at longer wavelengths, suggesting that deeper structures have a greater concentration.^42^

The reduced scattering coefficient, $\mu_{s}^{\prime }$, values of the dermis are noticeably higher than those of subcutaneous tissue, especially in the visible range, likely due to the higher density of collagen fibers and other microstructures in the dermis layer. As expected from theory, $\mu_{s}^{\prime }$ of both tissue types shows a general monotonic decrease in value with increasing wavelength.

### 3.2 Post-storage Change in Absorption

**Fig. 4:**
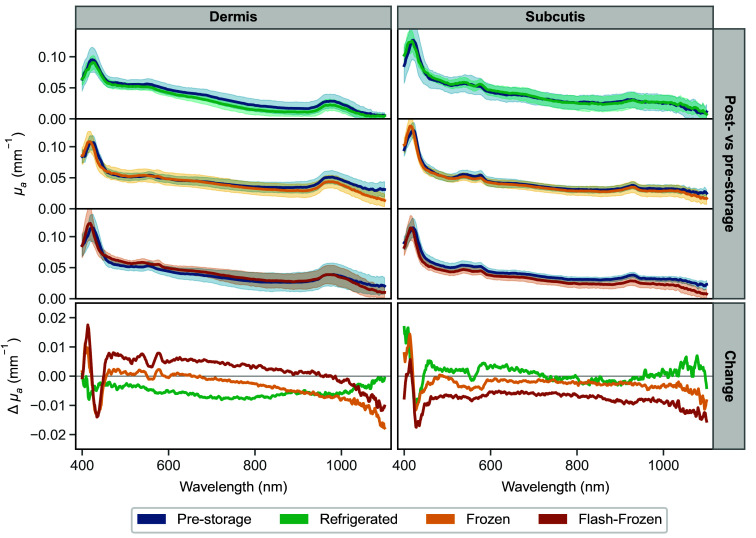
Change in the absorption coefficient, $\mu_{a}$, following storage of the dermis (left) and subcutaneous tissue (right): comparison of average pre- and post-storage $\mu_{a}$ for each storage condition (top), and the average absolute change from the pre-storage values (bottom). Axes are kept consistent, and shaded regions represent the standard deviation.

The comparison of the average absorption coefficient values pre- and post-storage for each storage condition is depicted in Fig. 4, with the values for the dermis on the left side, and those of the subcutaneous layer on the right. The top section shows the average $\mu_{a}$ measurement of refrigerated, frozen, and flash-frozen storage groups with the corresponding pre-storage measurement on the same plot. The bottom section displays the average change of the samples from the pre-storage $\mu_{a}$ for each storage condition.

Overall, the change in $\mu_{a}$ after the storage is small for both tissues, with dermis samples experiencing an average change of −0.005, −0.003, and $0.002\;{\mathrm{mm}}^{-1}$ and subcutaneous samples a change of 0.001, −0.003, and $-0.008\;{\mathrm{mm}}^{-1}$ for refrigerated, frozen, and flash-frozen groups, respectively. For reference, the average pre-storage $\mu_{a}$ is in the 0.019 to $0.107\;{\mathrm{mm}}^{-1}$ and 0.018 to $0.124\;{\mathrm{mm}}^{-1}$ ranges for the dermis and subcutis, respectively. A Wilcoxon signed-rank test was performed per wavelength on the pre- and post-storage optical absorption coefficient values to assess the effect of cold storage. The results indicated a significant median difference (p<0.05) in the Soret band region of the spectrum in frozen and flash-frozen groups, and Q band region in flash-frozen groups for both tissues. A significant difference was also indicated in the wavelengths above 1000 nm for frozen and flash-frozen tissues, and the majority of the spectrum for flash-frozen subcutaneous fat.

Some notable differences can be observed in the hemoglobin region of the dermis spectra. Prior to storage, the dermis $\mu_{a}$ spectrum exhibits features of deoxyhemoglobin, namely the position of the Soret peak at 424 nm and a single Q band. After storage, whereas there is no apparent change in Soret or Q band in the refrigerated group, both frozen and flash-frozen groups demonstrate a clear blue shift of the Soret band and emergence of the double Q bands that are characteristic of oxyhemoglobin. To a lesser degree, the post-storage $\mu_{a}$ values of the subcutaneous layer show a similar blue shift of the Soret band.

*In vivo*, the stratum corneum acts as a barrier to external gas exchange;^43^ however, the excised tissue is fully exposed to the residual air in the sample bags. The difference in the change in hemoglobin oxygenation between the refrigerated samples and the frozen or flash-frozen samples could be due to differences in the amount of time each group was exposed to air. Refrigerated samples were stored for ∼24  h, whereas the frozen and flash-frozen tissues were measured after 1 week. It is also possible the lower temperatures may have increased hemoglobin’s affinity for oxygen. Lower temperatures are known to cause a left shift in the hemoglobin saturation curve, resulting in a higher oxygen saturation for a given partial pressure of oxygen.^44^ It has also been previously suggested that hemolysis resulting from freezing may accelerate oxygen uptake due to the release of free hemoglobin into the plasma.^23^^,^^24^ Ultimately, each of these factors may to some degree be responsible for the observed change in oxidation state.

Aside from the region of the absorption spectrum dominated by hemoglobin, there is a small observable dip in the $\mu_{a}$ values of frozen and flash-frozen groups above 980 nm. This downward trend is possibly due to mild dehydration of the samples during longer storage in subzero temperatures. The dip is not present in the measurements of the refrigerated group, which was stored wrapped in gauze moistened with saline.

### 3.3 Post-storage Change in Scattering

**Fig. 5:**
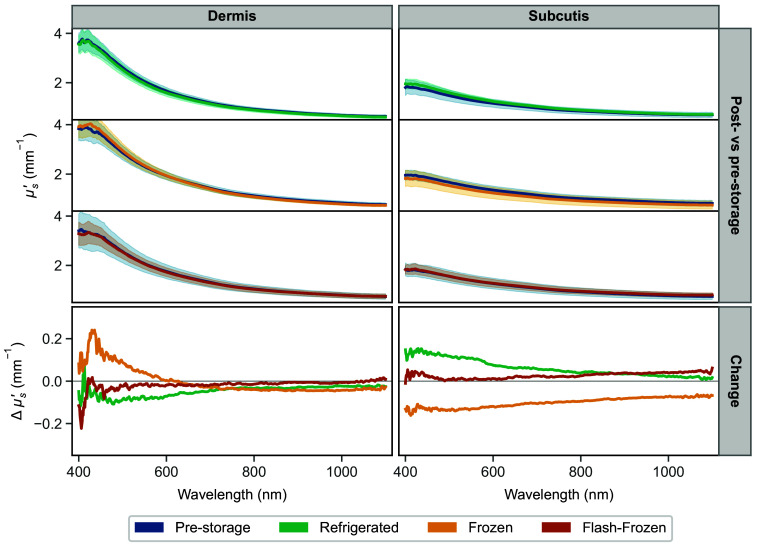
Change in the reduced scattering coefficient, $\mu_{s}^{\prime }$, following storage of the dermis (left) and subcutaneous tissue (right): comparison of average pre- and post-storage $\mu_{s}^{\prime }$ for each storage condition (top), and the average absolute change from the pre-storage values (bottom). Axes are kept consistent, and shaded regions represent the standard deviation.

The average reduced scattering coefficient values before and after storage for each storage condition, as well as the average change, are shown in Fig. 5. Similarly to the previous figure illustrating the changes in $\mu_{a}$, the $\mu_{s}^{\prime }$ values for the dermis and subcutaneous tissue are plotted on the left and right sides, respectively. The average $\mu_{s}^{\prime }$ post-storage and corresponding pre-storage measurement are displayed in the top section of the figure, whereas the bottom section shows the average change of the samples from the pre-storage value for each storage group.

The change in $\mu_{s}^{\prime }$ after the storage is small in both tissues: the average change in dermis samples is −0.047, −0.001, and $-0.017\;{\mathrm{mm}}^{-1}$, and in subcutaneous tissue, it is 0.062, −0.102, and $0.026\;{\mathrm{mm}}^{-1}$ for the refrigerated, frozen, and flash-frozen groups, respectively. For scale reference, the average pre-storage values of $\mu_{s}^{\prime }$ fall in the 0.7 to $3.7\;{\mathrm{mm}}^{-1}$ for the dermis and 0.7 to $1.9\;{\mathrm{mm}}^{-1}$ ranges for subcutaneous fat. For the reduced scattering coefficient, the results of a Wilcoxon signed-rank test indicated a significant difference (p<0.05) in the 500 to 850 nm range for subcutaneous fat following frozen storage, but not other groups.

Storage does not appear to have a pronounced effect on the scattering properties of the dermis and subcutaneous tissue, although loss of moisture during prolonged storage can impact them. Hydration differences during tissue sample preparation are known to have a large effect on coefficient measurements,^17^^,^^45^^,^^46^ and dehydration has been shown to be one of the possible mechanisms of tissue optical clearing.^47^ On the one hand, an increase in density due to dehydration would result in tighter packing of scattering particles as well as absorbers, increasing the probability of a scattering event. On the other hand, water evaporation is likely to increase the refractive index of the interstitial fluid or ground substance due to an increase in proteoglycan concentration, leading to a greater degree of index matching within the tissue (optical clearing), and thus a lower scattering probability. In addition, structural tissue damage due to ice crystal formation during slow freezing without cryoprotectants may also contribute to the reduction in scattering in the frozen group.

### 3.4 Assessment of Methods

This study improves upon the methodology of the goniospectrophotometry study performed by DeLisi et al. The spectrophotometry method was hindered by a low SNR, which can be attributed to an exceptionally low system throughput that required long integration times to produce viable data. It was not possible to sample at all scattering angles, increasing the uncertainty in the reflectance and transmittance measurements. Further, integrating the total reflectance and transmittance using the goniospectrophotometer required the use of a curve fit, which does not have a physical basis. Integrating spheres is an accepted standard for measuring tissue optical properties that do not require a curve fit because the total reflectance and transmittance from all angles are physically integrated by the sphere. As the present method collected a very large portion of light across the scattering angles, with the caveat that some light is lost through the ports, the throughput of the integrating sphere method is considerably higher. The method used in this study also benefits from extended NIR range silicon spectrometers, which provide adequate SNR out to 1100 nm without detector-related discontinuities.

Acquiring pre-storage measurements within a short time after tissue excision is a considerably time-consuming process. Half of each measurement day was devoted to subject preparation, biopsy, and tissue slicing. Measuring a single group of 10 samples took roughly 45 min, including calibration, tissue mounting, measurements, and storage preparation. Group sizes were limited to 10 due to the high cost of quality fused silica slides, and the requirement to clean slides between samples, which took up over half of the reported time.

A Wilcoxon signed-rank test was performed to evaluate the optical property differences following cold storage. However, the study presented here included a limited number of total samples due to the requirement of measuring freshly excised tissue. Given the limited data points, the reported findings should be supported by further studies. Future work would benefit from expanding the number of subjects and using a mixed-effects approach to assist in discriminating between subjects, measurement day, system calibration, and storage conditions.

We must acknowledge that both glass refractive index and Spectralon/Spectraflect^®^ reflectance vary with wavelength. The appropriate wavelength-specific values can be estimated through empirical formulae, such as the Sellmeier equation for the refractive index, or obtained from the manufacturer. In our analysis, the wavelength dependence was not considered because the primary objective was to compare pre- and post-storage measurements based on individual wavelengths, rather than focusing on absolute values.

It is worth noting that compression of soft tissue is known to affect its optical properties;^16^^,^^48^^–^^50^ however, it is quite difficult to account for pressure while mounting samples. It may be possible to circumvent this issue by using rigid shims to create a fixed distance between glass surfaces. However, the shims would need to be adjusted for each sample, and changes in the sample thickness are dependent on the storage mechanism. Even determining the sample thickness with a dial lens gauge can cause a small degree of compression. In the present study, we ensure that the pressure or compression force remains consistent for all samples by positioning the tissue between two glass slides in a simple setup without additional compression. The top slide merely rests on the sample, and the top integrating sphere’s position is slowly adjusted until the port is just flush with the glass slide, minimizing any potential applied pressure as much as possible.

## 4 Conclusion

In summary, we have examined the effect of cold storage on absorption and reduced scattering coefficients in the skin using a double integrating sphere system and IAD algorithm. Samples of porcine dermis and subcutaneous tissue were measured prior to storage within 2 h of biopsy and after storage by refrigeration at 4°C for 24 h, freezing at −18°C for 1 week, and flash freezing by immersion in liquid nitrogen with subsequent storage at −80°C for 1 week.

Results demonstrated a small change in absorption coefficient for both tissue types, with an average change of −0.005, −0.003, and $0.002\;{\mathrm{mm}}^{-1}$ in the dermis and 0.001, −0.003, and $-0.008\;{\mathrm{mm}}^{-1}$ in the subcutaneous tissue after refrigeration, freezing, and flash freezing, respectively. As noted in previous studies, the largest contributors to optical absorption differences pre- and post-storage are hemoglobin concentration and oxidation state. Storage notably affects the absorption near hemoglobin’s Soret and Q band peaks, especially in the dermis tissue. Specifically, the spectra indicate the presence of deoxyhemoglobin in pre-storage and refrigerated spectra, and oxyhemoglobin after frozen and flash-frozen storage. In comparison, a decrease in absorption coefficient after storage was reported previously, with different tissue under investigation,^24^^,^^25^ and a pre-storage baseline that was collected within minutes after animal euthanasia.^23^ Scattering appears to be robust to storage-induced changes, with negligible changes detected for both tissues. In the dermis, the reduced scattering coefficient decreased by an average of −0.05, −0.001, and $-0.02\;{\mathrm{mm}}^{-1}$ for refrigerated, frozen, and flash-frozen groups, whereas in subcutaneous tissue, it increased by 0.06 and $0.03\;{\mathrm{mm}}^{-1}$ with refrigeration and flash freezing, but decreased by $-0.1\;{\mathrm{mm}}^{-1}$ for the frozen group. Results of prior studies on different tissues^23^^–^^25^ indicated a small decrease of reduced scattering coefficient with subzero storage, and either a small decrease or increase with refrigeration, likely due to difference in tissue type or handling.

The findings of this study indicate that dermis and subcutaneous fat exhibit minimal change in optical absorption and reduced scattering coefficient values after refrigeration, freezing, or flash-freezing, compared with their pre-storage measurements. However, we establish the importance of accounting for the samples’ storage conditions when using values for the 400 to 600 nm region. Furthermore, the results provide additional context for the variability of published values of optical parameters and inform the selection of datasets used in computational models. In addition, the data presented here can help inform future researchers with their experimental design such that they can optimize collection and storage methodology.

## Supplementary Material



## Data Availability

The data used in this study is freely available upon reasonable request to the authors.
