# Supplementary material for: Effects of cold storage on double integrating sphere optical property measurements of porcine dermis and subcutaneous fat from 400 to 1100 nm
Source: J Biomed Opt. 2025 Jan 22;30(1):015001. doi: 10.1117/1.JBO.30.1.015001 (PMC11751729; doi:10.1117/1.JBO.30.1.015001)
Supplement: Supplementary file 1 [file JBO_030_015001_SD001.pdf]

Supplementary Materials:

Effects of cold storage on double integrating sphere optical properties measurements of porcine dermis and subcutaneous fat from 400 to 1100 nm

Maria A. T. Hoffman[a,+], Mark A. Keppler[a,b,+], Andrea L. Smith[a], Anjelyka Fasci[a,d], Matthew E. Macasadia[a], Amanda J. Tijerina[c], Robert Lyle Hood[d], Michael P. DeLisi[a], Joel N. Bixler[b,e*]

[a]SAIC, JBSA Fort Sam Houston, Texas 78234
[b]Texas A&M University, Department of Biomedical Engineering, College Station, Texas 77843
[c]Conceptual Mindworks, Inc., San Antonio, Texas 78234
[d]University of Texas at San Antonio, Department of Mechanical Engineering, San Antonio, Texas 78249
[e]Air Force Research Laboratory, JBSA Fort Sam Houston, Texas 78234
[+]These authors contributed equally to this work

1 Biomimic™ Tissue Phantom

Considerable effort has been taken to ensure system performance remained stable across all measurement days. The system baseline was tracked and compared to a NIST-traceable polyurethane Biomimic™ optical phantom (F0660, INO, Canada) before any tissue measurements were acquired. The Biomimic™ phantom is a hard highly stable incompressible material with some surface texture. Because the polyurethane sample was considerably larger than the tissue samples, it was necessary to acquire baseline measurements with sample port apertures of 12.7 mm instead of 6.35 mm port reducer caps used for tissue samples. The phantom was placed into the double integrating sphere system without glass slides. The manufacturer-provided values for the refractive index and the anisotropy factor were 1.511±0.006 and 0.620±0.015 respectively. The thickness of the standard was 4.934±0.004 mm as measured using a precision dial lens gauge (GA-725, Vigor, USA).

System stability analysis was performed through measurements of the Biomimic™ phantom, and are expressed as standard deviation values. The shot-to-shot (without sample repositioning)

uncertainty across the 400 to 1100 nm range was determined to be 7×10^{-4} mm^{-1} and 7.1×10^{-3} mm^{-1} for μ_a and μ_s' respectively. The day-to-day uncertainty over 7 days was determined to be 1.3×10^{-3} mm^{-1} for μ_a and 1.8×10^{-2} mm^{-1} for μ_s'.

2 Average absorption and reduced scattering coefficient values

The average pre- and post-storage values of absorption, μ_a, and reduced scattering, μ_s', coefficients are provided in Tables S1 and S2 respectively. The spectra cover the full 400 to 1100 nm range in steps of 10 nm. The asterisk (*) is used to indicate a significant difference from the corresponding pre-storage values, based on Wilcoxon signed-rank test ($p < 0.05$). Please note that the pre-storage values presented in these supplemental tables are averaged across all three storage groups.

Table S1: Average values of μ_a, mm^{-1} (*$p < 0.05$)

	Dermis				Subcutis			
λ, nm	Pre-storage	Refrigerated	Frozen	Flash Frozen	Pre-storage	Refrigerated	Frozen	Flash Frozen
400	0.079	0.064	0.086	0.085	0.091	0.102	0.103	0.083
410	0.091	0.078	0.101	0.112	0.110	0.123	0.127	0.106
420	0.106	0.088	0.108	0.120	0.122	0.123	0.127	0.110
430	0.101	0.089	0.089*	0.097*	0.104	0.104	0.094	0.082*
440	0.083	0.077	0.070*	0.077*	0.080	0.084	0.073	0.063*
450	0.068	0.064	0.063	0.070	0.068	0.075	0.063	0.055*
460	0.059	0.058	0.060	0.067*	0.062	0.069	0.059	0.052*
470	0.057	0.055	0.057	0.064*	0.059	0.065	0.057	0.049*
480	0.056	0.054	0.056	0.062*	0.056	0.063	0.054	0.047*
490	0.054	0.053	0.054	0.060*	0.054	0.060	0.052	0.046*
500	0.053	0.053	0.053	0.058*	0.052	0.058	0.050	0.044*
510	0.052	0.052	0.052	0.057*	0.051	0.055	0.049	0.043*
520	0.053	0.052	0.052	0.057*	0.052	0.057	0.049	0.044*
530	0.053	0.052	0.054	0.058*	0.055	0.058	0.051	0.046*
540	0.053	0.051	0.054	0.058*	0.055	0.059	0.052	0.047
550	0.054	0.052	0.053	0.057	0.054	0.056	0.050	0.046*
560	0.054	0.051	0.053	0.055	0.053	0.053	0.048	0.044*
570	0.051	0.048	0.052	0.055*	0.052	0.054	0.049	0.044*
580	0.049	0.045	0.052	0.055*	0.052	0.055	0.049	0.044*
590	0.048	0.044	0.049	0.050*	0.045	0.048	0.043	0.039
600	0.047	0.042	0.048	0.049	0.043	0.047	0.042	0.038
610	0.045	0.041	0.048	0.048*	0.042	0.045	0.041	0.036*
620	0.044	0.038	0.047	0.047*	0.041	0.044	0.040	0.036*
630	0.043	0.038	0.046	0.046	0.041	0.043	0.040	0.036

Continued on next page

Table S1: Average values of μ_a, mm^{-1}, continued

	Dermis				Subcutis			
λ, nm	Pre-storage	Refrigerated	Frozen	Flash Frozen	Pre-storage	Refrigerated	Frozen	Flash Frozen
640	0.043	0.036	0.046	0.046	0.041	0.042	0.039	0.035
650	0.042	0.036	0.045	0.045	0.040	0.041	0.039	0.035*
660	0.042	0.034	0.045	0.044	0.040	0.042	0.039	0.035*
670	0.041	0.033	0.044	0.044	0.039	0.040	0.038	0.035
680	0.040	0.032	0.043	0.043	0.039	0.039	0.038	0.034
690	0.039	0.030	0.042	0.042	0.038	0.037	0.036	0.033*
700	0.038	0.028	0.041	0.041	0.037	0.036	0.035	0.032
710	0.037	0.027	0.040	0.040	0.036	0.034	0.035	0.030
720	0.036	0.025	0.039	0.038	0.035	0.033	0.034	0.030
730	0.035	0.023	0.038	0.038	0.034	0.031	0.033	0.029*
740	0.034	0.022	0.038	0.037	0.033	0.030	0.032	0.028*
750	0.033	0.020	0.036	0.035	0.032	0.028	0.031	0.028
760	0.032	0.019	0.036	0.034	0.032	0.028	0.031	0.027
770	0.031	0.018	0.035	0.034	0.031	0.026	0.030	0.026*
780	0.030	0.016	0.034	0.033	0.030	0.026	0.029	0.025*
790	0.029	0.015	0.033	0.031	0.030	0.026	0.029	0.025*
800	0.029	0.014	0.033	0.031	0.029	0.025	0.029	0.024*
810	0.028	0.014	0.032	0.031	0.030	0.025	0.029	0.024*
820	0.028	0.013	0.031	0.030	0.030	0.024	0.028	0.024*
830	0.028	0.012	0.031	0.029	0.030	0.024	0.029	0.024
840	0.027	0.012	0.031	0.029	0.030	0.025	0.028	0.024*
850	0.027	0.012	0.031	0.029	0.029	0.024	0.028	0.024*
860	0.027	0.012	0.030	0.029	0.029	0.023	0.028	0.023*
870	0.027	0.011	0.030	0.028	0.029	0.024	0.027	0.023*

Continued on next page

Table S1: Average values of μ_a, mm^{-1}, continued

	Dermis				Subcutis			
λ, nm	Pre-storage	Refrigerated	Frozen	Flash Frozen	Pre-storage	Refrigerated	Frozen	Flash Frozen
880	0.026	0.011	0.029	0.028	0.030	0.025	0.028	0.023*
890	0.027	0.011	0.029	0.027	0.031	0.025	0.029	0.024*
900	0.027	0.011	0.029	0.028	0.031	0.026	0.030	0.025*
910	0.027	0.011	0.029	0.028	0.033	0.027	0.032	0.026*
920	0.027	0.011	0.029	0.029	0.035	0.029	0.033	0.028*
930	0.028	0.012	0.030	0.030	0.036	0.030	0.035	0.029*
940	0.030	0.013	0.032*	0.031	0.033	0.028	0.032	0.027*
950	0.034	0.017	0.036	0.034	0.031	0.026	0.029	0.025*
960	0.039	0.021	0.042	0.038	0.031	0.028	0.029	0.024*
970	0.040	0.022	0.044	0.039	0.031	0.027	0.029	0.024*
980	0.040	0.022	0.044	0.039	0.031	0.027	0.029	0.024*
990	0.039	0.021	0.042	0.036	0.030	0.027	0.028	0.024*
1000	0.037	0.018	0.039	0.034	0.030	0.027	0.029	0.023*
1010	0.034	0.017	0.036*	0.032	0.030	0.025	0.029	0.023*
1020	0.031	0.013	0.033*	0.029	0.030	0.026	0.029	0.022*
1030	0.028	0.010	0.031*	0.026	0.029	0.023	0.028	0.021*
1040	0.026	0.009	0.027*	0.022	0.028	0.025	0.027	0.019*
1050	0.024	0.007	0.024*	0.019	0.026	0.022	0.025	0.016*
1060	0.022	0.005	0.021*	0.016	0.025	0.018	0.023	0.015*
1070	0.021	0.004	0.019*	0.015	0.023	0.018	0.022	0.014*
1080	0.020	0.005	0.017*	0.013*	0.019	0.015	0.019*	0.011*
1090	0.021	0.005	0.016*	0.011	0.020	0.012	0.018*	0.008*
1100	0.020	0.005	0.013*	0.010	0.021	0.007	0.017*	0.008*

Table S2: Average values of $\mu\prime_s$, mm^{-1} (*$p < 0.05$)

	Dermis				Subcutis			
λ, nm	Pre-storage	Refrigerated	Frozen	Flash Frozen	Pre-storage	Refrigerated	Frozen	Flash Frozen
400	3.615	3.556	3.919	3.284	1.876	1.954	1.822	1.828
410	3.652	3.726	3.932	3.239	1.868	1.952	1.800	1.814
420	3.645	3.683	4.009	3.322	1.871	1.949	1.820	1.852
430	3.533	3.526	3.992	3.287	1.826	1.912	1.786	1.813
440	3.475	3.413	3.868	3.215	1.807	1.879	1.771	1.791
450	3.383	3.280	3.751	3.162	1.777	1.834	1.723*	1.760
460	3.255	3.126	3.597	3.019	1.737	1.785	1.690*	1.719
470	3.096	2.954	3.421	2.886	1.695	1.731	1.653*	1.678
480	2.964	2.802	3.268	2.776	1.655	1.684	1.603*	1.643
490	2.821	2.659	3.105	2.638	1.615	1.641	1.562*	1.596
500	2.685	2.522	2.952	2.544	1.576	1.595	1.529*	1.561
510	2.565	2.398	2.812	2.435	1.538	1.549	1.492*	1.529
520	2.450	2.274	2.678	2.322	1.504	1.509	1.456*	1.495
530	2.339	2.165	2.550	2.226	1.467	1.470	1.425*	1.463
540	2.244	2.064	2.451	2.141	1.433	1.431	1.390*	1.424
550	2.151	1.972	2.344	2.058	1.403	1.399	1.360*	1.401
560	2.070	1.881	2.255	1.976	1.373	1.367	1.329*	1.374
570	1.993	1.798	2.161	1.913	1.345	1.336	1.303*	1.344
580	1.920	1.726	2.087	1.849	1.320	1.304	1.279*	1.320
590	1.856	1.656	2.006	1.786	1.296	1.268	1.256*	1.299
600	1.792	1.596	1.939	1.727	1.273	1.242	1.234*	1.278
610	1.729	1.536	1.872	1.678	1.250	1.215	1.209*	1.255
620	1.676	1.482	1.805	1.622	1.230	1.195	1.192	1.233
630	1.622	1.433	1.749	1.579	1.211	1.172	1.172*	1.218

Continued on next page

Table S2: Average values of μ'_s, mm^{-1}, continued

	Dermis				Subcutis			
λ, nm	Pre-storage	Refrigerated	Frozen	Flash Frozen	Pre-storage	Refrigerated	Frozen	Flash Frozen
640	1.574	1.384	1.688	1.533	1.191	1.150	1.154*	1.199
650	1.529	1.337	1.637	1.490	1.170	1.129	1.137*	1.182
660	1.485	1.295	1.584	1.448	1.151	1.111	1.120*	1.163
670	1.440	1.254	1.542	1.409	1.132	1.092	1.104*	1.145
680	1.397	1.216	1.485	1.369	1.114	1.074	1.087*	1.127
690	1.357	1.175	1.442	1.328	1.094	1.054	1.066*	1.110
700	1.313	1.141	1.392	1.290	1.076	1.034	1.047*	1.091
710	1.274	1.104	1.351	1.255	1.056	1.013	1.026*	1.072
720	1.242	1.076	1.308	1.222	1.038	0.994	1.009*	1.054
730	1.209	1.046	1.268	1.194	1.022	0.978	0.993*	1.037
740	1.176	1.018	1.237	1.167	1.008	0.960	0.981*	1.024
750	1.149	0.995	1.206	1.138	0.992	0.947	0.966*	1.008
760	1.122	0.968	1.173	1.116	0.980	0.933	0.951*	0.996
770	1.099	0.951	1.148	1.093	0.968	0.922	0.941*	0.983
780	1.078	0.932	1.123	1.069	0.957	0.910	0.931*	0.975
790	1.057	0.910	1.099	1.049	0.949	0.896	0.922*	0.966
800	1.038	0.891	1.077	1.033	0.938	0.888	0.912*	0.956
810	1.019	0.875	1.055	1.017	0.926	0.880	0.904*	0.947
820	1.001	0.858	1.035	1.000	0.918	0.871	0.895*	0.940
830	0.984	0.845	1.016	0.983	0.908	0.862	0.886*	0.932
840	0.968	0.831	0.999	0.968	0.900	0.851	0.879*	0.923
850	0.952	0.817	0.980	0.951	0.891	0.844	0.873*	0.914
860	0.936	0.803	0.962	0.938	0.883	0.837	0.865	0.906
870	0.921	0.787	0.943	0.925	0.874	0.827	0.855*	0.901

Continued on next page

Table S2: Average values of $\mu\prime_s$, mm^{-1}, continued

	Dermis				Subcutis			
λ, nm	Pre-storage	Refrigerated	Frozen	Flash Frozen	Pre-storage	Refrigerated	Frozen	Flash Frozen
880	0.907	0.774	0.930	0.912	0.867	0.819	0.850*	0.890
890	0.894	0.764	0.914	0.900	0.859	0.812	0.842*	0.884
900	0.880	0.752	0.901	0.889	0.851	0.804	0.836	0.878
910	0.867	0.739	0.887	0.875	0.843	0.796	0.829	0.869
920	0.853	0.727	0.870	0.862	0.835	0.788	0.822	0.861
930	0.842	0.717	0.858	0.852	0.828	0.781	0.815*	0.853
940	0.832	0.705	0.847	0.843	0.822	0.773	0.810	0.846
950	0.825	0.698	0.837	0.834	0.816	0.768	0.805	0.842
960	0.815	0.690	0.828	0.826	0.811	0.761	0.799	0.835
970	0.807	0.683	0.817	0.817	0.806	0.757	0.793	0.831
980	0.796	0.674	0.805	0.808	0.799	0.749	0.787*	0.824
990	0.787	0.668	0.797	0.800	0.794	0.745	0.782	0.821
1000	0.777	0.656	0.785	0.792	0.787	0.739	0.775	0.814
1010	0.766	0.650	0.776	0.782	0.783	0.734	0.770	0.809
1020	0.760	0.643	0.767	0.775	0.778	0.730	0.764*	0.806
1030	0.753	0.638	0.760	0.767	0.776	0.724	0.763	0.804
1040	0.745	0.635	0.754	0.763	0.771	0.719	0.758	0.801
1050	0.739	0.627	0.750	0.755	0.769	0.720	0.754	0.799
1060	0.731	0.623	0.741	0.755	0.767	0.718	0.752	0.797
1070	0.728	0.618	0.734	0.752	0.762	0.711	0.746*	0.796
1080	0.725	0.618	0.731	0.752	0.761	0.710	0.750*	0.791
1090	0.721	0.615	0.735	0.746	0.761	0.710	0.744	0.790
1100	0.719	0.613	0.735	0.749	0.759	0.719	0.744	0.805*

List of Tables

S1 Average values of μ_a, mm^{-1} ($*p < 0.05$)

S1 Average values of μ_a, mm^{-1}, continued

S1 Average values of μ_a, mm^{-1}, continued

S2 Average values of $\mu\prime_s$, mm^{-1} ($*p < 0.05$)

S2 Average values of $\mu\prime_s$, mm^{-1}, continued

S2 Average values of $\mu\prime_s$, mm^{-1}, continued
